# What, Where, and How to Collect Real-World Data and Generate Real-World Evidence to Support Drug Reimbursement Decision-Making in Asia: A reflection Into the Past and A Way Forward

**DOI:** 10.34172/ijhpm.2023.6858

**Published:** 2023-03-06

**Authors:** Sarin Kc, Lydia Wenxin Lin, Diana Beatriz Samson Bayani, Yaroslava Zemlyanska, Amanda Adler, Jeonghoon Ahn, Kelvin Chan, Dechen Choiphel, Anne Julienne Genuino-Marfori, Brendon Kearney, Yuehua Liu, Ryota Nakamura, Fiona Pearce, Shankar Prinja, Raoh-Fang Pwu, Arsul Akmal Shafie, Binyan Sui, Auliya Suwantika, Sean Tunis, Hui-Min Wu, John Zalcberg, Kun Zhao, Wanrudee Isaranuwatchai, Yot Teerawattananon, Hwee-Lin Wee

**Affiliations:** ^1^Health Intervention and Technology Assessment Program (HITAP), Ministry of Health, Nonthaburi, Thailand; ^2^Saw Swee Hock School of Public Health, National University of Singapore (NUS), Singapore, Singapore; ^3^The Oxford Centre for Diabetes, Endocrinology, and Metabolism, University of Oxford, Oxford, UK; ^4^Ewha Womans University, Seoul, South Korea; ^5^Sunnybrook Odette Cancer Centre, Toronto, ON, Canada; ^6^Sunnybrook Research Institute, Toronto, ON, Canada; ^7^Canadian Centre for Applied Research in Cancer Control, Toronto, ON, Canada; ^8^Essential Medicine and Technology Division, Department of Medical Services, Ministry of Health, Thimphu, Bhutan; ^9^Health Technology Assessment Unit, Department of Health, Quezon City, Philippines; ^10^Faculty of Medicine, University of Adelaide, Adelaide, SA, Australia; ^11^Health Policy Advisory Committee on Technology, Brisbane, QLD, Australia; ^12^China Health Technology Assessment Centre, National Health Development Research Centre, Ministry of Health, Beijing, China; ^13^Hitotsubashi Institute for Advanced Study, Hitotsubashi University, Tokyo, Japan; ^14^Agency for Care Effectiveness, Ministry of Health, Singapore, Singapore; ^15^Department of Community Medicine and School of Public Health, Post Graduate Institute of Medical Education and Research, Chandigarh, India; ^16^Taiwan National Hepatitis C Program Office, Ministry of Health and Welfare, Taipei, Taiwan; ^17^Discipline of Social and Administrative Pharmacy, School of Pharmaceutical Sciences, Universiti Sains Malaysia, Penang, Malaysia; ^18^Department of Pharmacology and Clinical Pharmacy, Faculty of Pharmacy, Universitas Padjadjaran, Sumedang, Indonesia; ^19^Center for Medical Technology Policy (CMTP), Baltimore, MD, USA; ^20^Cancer Research Program, School of Public Health and Preventive Medicine, Monash University, Melbourne, VIC, Australia; ^21^Department of Medical Oncology, Alfred Hospital, Melbourne, VIC, Australia; ^22^Centre for Excellence in Economic Analysis Research, St. Michael’s Hospital, Toronto, ON, Canada; ^23^Institute of Health Policy, Management and Evaluation, University of Toronto, Toronto, ON, Canada; ^24^Department of Pharmacy, Faculty of Science, National University of Singapore (NUS), Singapore, Singapore

**Keywords:** Asia, Cost-Effectiveness Analysis, Health Technology Assessment, Real-World Data, Real-World Evidence, Reimbursement

## Abstract

**Background:** Globally, there is increasing interest in the use of real-world data (RWD) and real-world evidence (RWE) to inform health technology assessment (HTA) and reimbursement decision-making. Using current practices and case studies shared by eleven health systems in Asia, a non-binding guidance that seeks to align practices for generating and using RWD/RWE for decision-making in Asia was developed by the **REAL **World Data **I**n A**S**ia for H**E**alth Technology Assessment in Reimbursement (REALISE) Working Group, addressing a current gap and needs among HTA users and generators.

**Methods:** The guidance document was developed over two face-to-face workshops, in addition to an online survey, a face-to-face interview and pragmatic search of literature. The specific focus was on what, where and how to collect RWD/ RWE.

**Results:** All 11 REALISE member jurisdictions participated in the online survey and the first in-person workshop, 10 participated in the second in-person workshop, and 8 participated in the in-depth face-to-face interviews. The guidance document was iteratively reviewed by all working group members and the International Advisory Panel. There was substantial variation in: (*a*) sources and types of RWD being used in HTA, and (*b*) the relative importance and prioritization of RWE being used for policy-making. A list of national-level databases and other sources of RWD available in each country was compiled. A list of useful guidance on data collection, quality assurance and study design were also compiled.

**Conclusion:** The REALISE guidance document serves to align the collection of better quality RWD and generation of reliable RWE to ultimately inform HTA in Asia.

## Background

 Real-world data (RWD) in healthcare is data collected during routine delivery of healthcare from several sources such as electronic medical records (EMRs), claims and billing activities, registries, and patient-generated data using digital tools.^1,2^ Evidence derived from extracting and analysing these data through, for example, observational studies or pragmatic trials, is known as real-world evidence (RWE).^2,3^ RWE is already utilised globally in healthcare decision-making from market authorisation and coverage decisions of medical products to impact evaluation.^4,5^ Research in the field continues to evolve and it is expected that new processes and global standards will emerge as more data and information become available on the use of RWE.^5^ RWD can play an instrumental role in Asia while making context-specific reimbursement decisions for several reasons. Asians are often under-represented in pivotal randomised controlled trials (RCTs).^6^ This problem is further compounded by the fact that although 60% of the global population lives in Asia, only 17% of global clinical trials are conducted in the region,^7,8^ owing to financial and human capacity barriers, different ethical and regulatory systems, a lack of research environment in certain jurisdictions, and operational challenges.^9^ Thus, RCTs or observational studies conducted outside of Asia may not sufficiently report the expected health outcomes of Asian populations, due to differences in healthcare infrastructure and clinical practices, ethics and judicial systems compared to other regions, as well as differences in sociocultural environment and genetic makeup between Caucasians and Asians.^10^

 Using RWE to estimate the benefits and risks of therapies in Asian populations can also help to contextualise health economic models to different local settings. In many Asian jurisdictions, such as China, India, Indonesia, Malaysia, the Philippines, Singapore, and Thailand, reimbursement decisions are currently made several years after regulatory approval and market entry, allowing sufficient time to collect RWD. For example, drugs prescribed by physicians before reimbursement decisions are made are paid for either out-of-pocket or through private insurance. In this case, RWD can provide more certainty regarding the effectiveness of technologies in the local setting and can inform the judicious use of new technologies recommended for reimbursement. Other Asian health systems, such as in Japan, Taiwan, and South Korea, make their reimbursement decisions concurrently with or closely after regulatory approval. In such jurisdictions, RWE is often used to re-assess initial funding decisions including the adjustment of price, and hence can help reduce uncertainty in decision-making and outcomes.^11,12^

 It is important to understand where Asia stands in using RWD to assess and create an environment where they can be effectively translated into RWE to inform decisions. The **REAL** World Data **I**n A**S**ia for H**E**alth Technology Assessment in Reimbursement **(**REALISE**)** initiative was established in 2019 as a collaboration between global experts, leaders from Asian health technology assessment (HTA) agencies, and academia from the Asia-Pacific region to understand how RWD and RWE currently inform reimbursement decisions, and to recommend ways to collect and use this information in a consistent and scientifically robust manner.The work was packaged into three thematic areas: (1) scenarios under which RWD is appropriate; (2) what types of RWD to collect, where and how to collect them; and (3) how to translate RWD into RWE. Subsequently, a landscape analysis of RWD for reimbursement decisions in Asia^13^ and a non-binding guidance document^14^ addressing the three themes were published in 2020. Based on these two documents, we learned that all 11 jurisdictions (Bhutan, China, India, Indonesia, Japan, Malaysia, the Philippines, Singapore, South Korea, Taiwan, and Thailand) collaborating under REALISE accepted using RWE in their HTA dossiers, usually as supplementary evidence. Only Bhutan, China, Malaysia, and the Philippines did not require justification for using RWE in their evaluations.^13^ Meanwhile, only South Korea had some guidance on the minimum standards for collecting and submitting RWD and RWE for HTAs.^13^ While there was consensus that country-specific guidance on the use of RWD and RWE may not be necessary as there are already several guidelines published for Europe and the United States which are generalisable to other settings, we considered that a consolidated document with insights from the Asian context would be beneficial to further the use of RWD and RWE in developing health policy in the region.

 In this paper, we present the findings from our work on the second theme of the REALISE project which describe the types of RWD, their sources, and methods of collection in the Asian context. We then highlight issues related to the use of RWD and provide recommendations to address them.

## Methods

 REALISE was initiated at an in-person meeting during the 8th HTAsiaLink Conference (https://www.htasialink.org/) held in South Korea in April 2019, where the scope of the project was discussed. The meeting complemented a 16-question online needs assessment survey completed by project members representing different Asian jurisdictions. Findings from the first meeting and the results from the survey about the use of RWD/RWE to inform HTAs in Asia have been published, and led to the development of three thematic focus areas, namely: (1) when to use RWD and RWE; (2) what types of RWD to collect, where to collect them, and how to collect them; and (3) how to translate RWD into RWE.^13^ To address these themes systematically, the REALISE project team agreed that there was a need to produce a guidance document for Asia.

 The second thematic area (what, where, and how to collect RWD), which is the subject of this article, was discussed at a second in-person meeting held in Singapore in October 2019. We conducted a facilitated, world-café^15^ style session comprising three topics (what type of RWD to collect, where, and how to collect them) with 10 (out of 11) REALISE members to consolidate experiences from their different jurisdictions. Data collected during this world-café session can be found in Supplementary file 1.

 In addition, hour-long one-on-one interviews were conducted with 8 (out of 11) members to supplement the information collected during the world café session. Members were asked to answer 3 questions: (*i*) what examples of RWD are being collected in your country?; (*ii*) what are the sources of those RWD in your country?; and (*iii*) what methods are being used to translate those RWD into RWE?

 Information from the world-café session and one-on-one interviews was summarised, then reviewed and endorsed by all 11 REALISE members before being incorporated into the guidance document. In addition, we conducted a pragmatic search of literature based on the knowledge of REALISE members to identify guidance and country-specific examples on RWD and RWE. Relevant information was incorporated into the REALISE guidance document. We conducted a public consultation on the draft guidance document (for all three themes) through the REALISE website (https://hiper.nus.edu.sg/realise-guidance) in October 2020 before publishing the final guidance (version 1.1) in November 2020.

## Results

###  What Real-World Data to Collect? 

 The types of RWD used by REALISE members have been classified into four broad categories: (*i*) disease context – which includes incidence, prevalence, and transition probabilities; (*ii*) patient population – which includes socioeconomic, demographic, insurance and medical history; (*iii*) intervention and comparator – which includes dosage and regimens, continuation and discontinuation from treatment, and adherence; and (*iv*) outcomes – including safety, effectiveness, patient-reported outcomes (PROs), and costs. The RWD data sources identified in each jurisdiction included disease-specific or other registries, claims databases, health surveys, EMRs, and wearables and personal tracking devices. A summary of what type of RWD are used by each jurisdiction (proxied by responses from REALISE members) and which sources are typically used to collect RWD are summarised in Table. The information presented is not intended to be a comprehensive list and only highlights the types of RWD and their sources that have been used by REALISE members in their previous HTAs.

**Table:** Types of Real-World Data Collected by REALISE Members to Inform Health Technology Assessments, and Their Sources

**Types of RWD**	**Sources of RWD**				
	**Disease and Other Registries**	**Claims Database**	**Health Surveys**	**Electronic Medical Records**	**Wearables, Personal Tracking Devices **
1. Disease context (incidence, prevalence, transitional probabilities)	IN, JP, KR, MY, SG, TW, TH	IN, JP, KR, MY, SG, TW, TH	IN, JP, KR, MY, SG, TW, TH	IN, JP, KR, MY, SG, TW	TW
2. Patient population (age, sex, ethnicity, geographical location, income, education, insurance, medical history)	IN, JP, MY, SG, TW, TH	IN, JP, KR, MY, SG, TW, TH	IN, JP, KR, MY, SG, TW, TH	IN, JP, MY, SG, TW, TH	IN, JP, MY, SG, TW
3. Intervention & comparator (dosage, treatment continuation, waning of effect, discontinuation rates and reasons for discontinuation)	IN, JP, KR, MY, SG, TW, TH	IN, JP, KR, MY, SG, TW		IN, JP, KR, MY, SG, TW, TH	IN, JP, MY, SG, TW
Adherence (direct measures of drug levels, prescription refill rates, clinician assessments)	TW, TH	IN, JP, KR, MY, SG, TW	IN, JP, MY, SG, KR	IN, JP, KR, MY, SG, TW, TH	IN, JP, MY, SG, TW
4. Outcomes					
Safety (adverse drug events)	IN, JP, KR, MY, SG, TW, TH	IN, KR, MY, SG, TW		IN, JP, KR, MY, SG, TW, TH	TW
Effectiveness (surrogate or final outcomes for eg, mortality)	IN, JP, KR, MY, SG, TW, TH	IN, JP, KR, MY, SG, TW, TH		IN, JP, KR, MY, SG, TW, TH	IN, JP, MY, SG, TW
Patient reported outcomes (generic or disease specific measures)	IN, JP, KR, MY, SG, TW, TH		IN, JP, KR, MY, SG, TW, TH	TH	IN, JP, MY, SG, TW
Cost (cost or resource use)		IN, JP, KR, MY, SG, TW, TH	KR, TH	IN, JP, KR, MY, SG, TW, TH	

Abbreviations: IN, India; JP, Japan; KR, Korea; MY, Malaysia; SG, Singapore; TW, Taiwan; TH, Thailand; RWD, real-world data; REALISE, REAL World Data In ASia for HEalth Technology Assessment in Reimbursement.

**Figure 1:**
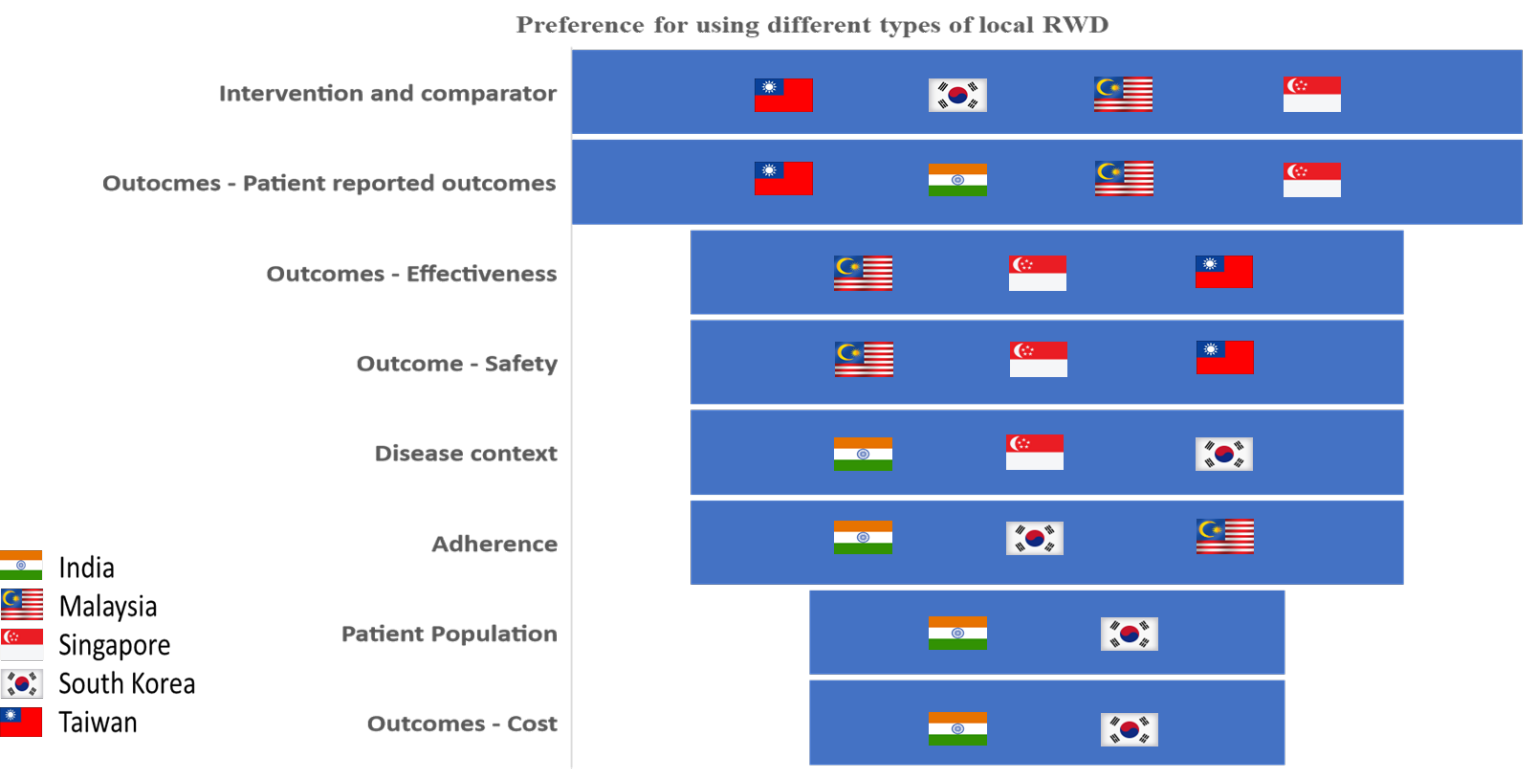
Types of Real-World Data That Should Be Context-Specific Ranked in Order. Abbreviation: RWD, real-world data.

 When considering members’ preferences for using certain types of local RWD in their HTAs, PROs emerged as the most common type (4 votes) followed by RWD about intervention and comparator, then outcomes – effectiveness, outcomes – safety, disease context, and adherence which received 3 votes each, followed by patient population and outcomes – cost which received 2 votes each (Figure 1).

###  Where and How to Collect Real-World Data? 

 Typical sources of RWD used by REALISE members are reported in Table. Members highlighted that they employed study designs such as observational studies and pragmatic clinical trials to extract and translate RWD into RWE for their local jurisdictions. A list of guidance on study design, optimal data collection, and quality assurance as well as some jurisdiction specific examples of how RWD collected from these sources have translated into policies are presented in the REALISE guidance. In response to public feedback,^16^ the draft REALISE guidance document was amended to also summarise the benefits and limitations of each data source identified, and incorporate good practices for collecting RWD and reporting results from observational studies and pragmatic clinical trials.

 Despite extensive use of registries, members highlighted several common hurdles in their use. The National Institute of Health (NIH) Centers for Disease Control and Prevention (KCDC) in Korea accept registration for clinical research in their Clinical Research Information Service system (https://cris.nih.go.kr). Publicly funded research, including many disease registries funded by KCDC are available, but most of them are considered investigator-initiated rather than public as researchers view the registries as their own. Public access to disease registries is therefore limited and potentially requires the individual to know the researchers or have contacts in the NIH/KCDC. A similar situation occurs in Japan whose registries have been implemented by medical societies and parties and are hence researcher-owned. Japanese registries are not allowed for HTA use. National registries in Singapore produce standard public reports of aggregated outcomes periodically, but they often have limited use as evidence to inform reimbursement decisions, given the data is not disaggregated and may not be relevant to inform estimates for subgroups or disease types. Several disease and health relevant registries (eg, births and deaths) are hosted by different public health institutions or other ministries which exacerbates the issue of data access and linkages.

 Members highlighted retrospective billing and claims databases often bring issues of data quality (missing data, unintentional miscoding, and intentional miscoding or ‘upcoding’), and may not contain all variables of interest (eg, symptoms, health status). Further, there is also often a lack of distinction between cost and charges, which can be an issue if claims data does not represent the economic value of resources used to provide services, and is influenced by monopolies or monopsony, then its utility for costing as part of a cost-effectiveness analysis is limited. Similar issues were reported with EMR data which are often incomplete, inaccurate, and requires cleaning before analysis. Health surveys were thought to be useful and additional source of RWD, especially disease or topic specific information is lacking either for a target sample or for the entire population. However, issues of subjectivity, and recall bias from respondents, and the lack of control over degree of data disaggregation were considered seen as common challenges by members while extracting RWD relevant to reimbursement from health surveys. Despite the promise of new technologies, extracting and using RWD from wearables and personal tracking devices remained a challenge for members due to lack of established accuracy, usability, and robustness. Therefore, use of RWD from such sources for regulatory or reimbursement decisions remains uncommon worldwide. Nonetheless, this was identified as a promising source of RWD and partnerships with the private sector (who are primary data custodians of such data) was considered necessary to future use.

## Discussion and Recommendations

 Based on our findings when developing the REALISE guidance document, it is evident that jurisdictions in Asia use RWD to produce RWE to inform reimbursement policies in different capacities. Focusing on the types and sources of RWD, depicted in Table, we noted key differences across jurisdictions. First, researchers in each country often accessed several data sources to collect the same type of RWD. For example, Malaysia used five sources to extract RWD on the patient population and we observed this for all other jurisdictions for different types of RWD. This highlights the current gaps and inefficiencies in data sharing across multiple agencies within each jurisdiction. For example, Thailand has three different health insurance schemes which are managed by different agencies. Therefore, certain RWD (even if they are the same type) need to be accessed from different sources. While this may be conducive to managing different schemes, it does point to fragmented data sources within health systems, incomplete data, or lack of standardising and integrating data.^17^ However, Thailand recognised this issues and launched the ‘Big Rock 1’ initiative, and is taking steps to link RWD within and outside the health sector.^18^ In Singapore, a white paper on responsible data sharing was jointly developed by the Precision Public Health Asia Society and the Saw Swee Hock School of Public Health.^19^ The authors advocated for establishing a data sharing framework, which will comprise: (*i*) a data sharing strategy, (*ii*) technical and technological capacity, (*iii*) regulatory and legal capacity, and (*iv*) an approach to operationalising data sharing. HTA agencies in Asia should contribute to developing data sharing framework as there are clear mutual benefits to be gained.

 Second, not all sources of RWD were used uniformly by jurisdictions to inform HTAs. For example, only Taiwan consistently used RWD from wearables and personal tracking devices. Similarly, not all jurisdictions consistently used EMRs to collect RWD. These differences may reflect the varying levels of maturity of each health system, and differences in capacity and incentives to capture RWD, as well as lack of access to different data sources.^20^ EMRs, if used properly, have the potential to collect most of the RWD we list in this paper, thereby reducing inefficiency in collecting data and eliminating the need to set up new disease specific registries, use claims databases to gather non-cost related data, or conduct ad-hoc surveys.^17,21^

 The format, completeness, and quality of RWD across different sources can vary significantly, and appropriate curation and validation are needed. In Asia, national standardisation of RWD variables between sources can and should be an important consideration among jurisdictions that are already making plans for country wide EMR systems (eg, Bhutan, China). Beneficiaries of standardising and harmonising data into EMRs include physicians, nurses, and researchers (RWD collectors and generators), health system managers (RWD custodians), researchers (RWE generators), and policy-makers (RWE consumers). Alternatively, stakeholders could assess the costs and benefits of accessing RWD from different sources in their settings and prioritise resources to those data sources. Incentives need to be developed for physicians and other providers, healthcare systems, payers, and patients to become invested stakeholders in the development and use of RWD and RWE.^22^ These include financial incentives from public and private insurance payers for quicker processing of reimbursement claims if accurate data are captured by the EMR; for hospitals if they submit laboratory and test results efficiently; for reporting on patient outcomes; providing RWD for studies; and for further adoption of payments, based on outcomes.^23^ Non-financial incentives include ensuring that RWD meets research needs and has clear value for those who collect them.

 Third, jurisdictions tended to vary in the way that they ranked the importance to different types of RWD with patient reported outcomes being the most preferred type of RWD, depicted in Figure 1. This may reflect the value attached to different types of RWD and their sources by researchers and policy-makers in those jurisdictions. These differences highlight that all RWD and their sources which could be used for HTA were not being used. This is not an issue if researchers and HTA agencies collectively decided to prioritise certain RWD depending on the context of the disease. However, it is an issue if they were not used due to structural or capacity barriers mentioned earlier. Furthermore, our initial findings from REALISE^13^ highlighted that (*i*) not all jurisdictions require justification for using RWD in HTA, and (*ii*) only South Korea (in 2019) had guidance or standards on using RWD for HTA. These observations are not conducive for using RWD for research.^24^ Addressing these issues may require explicitly outlining and standardising processes where the use of each type of RWD (depending on disease context), their sources, and methods to translate them into evidence are clearly outlined within the existing HTA decision-making framework. This sentiment is shared by the HTA community^25^ and the public in their feedback on the REALISE guidance document.^16^

###  Using HTA Process to Systematically Use RWD to Inform Decisions for Health Policy

 There are ongoing efforts to create a framework to incorporate RWE into decisions such as the Canadian Real-world Evidence for Value of Cancer Drugs.^26^ In the United States, the Food and Drug Administration published a preliminary framework for regulatory use of RWE in December 2018, with a focus on label expansion and changes, adding or modifying indications, as well as adding new populations and comparative effectiveness safety or cost information.^27^ While this framework has been established it has not yet been applied, and efforts are currently focused on demonstration projects with a number of institutions.^28^ Concurrently, the Institute for Clinical and Economic Review has published a white paper outlining the opportunities, challenges and limitations of RWE that need to be addressed while considering its use to inform insurer coverage decisions.^4^ European HTA organisations and payers also use RWE, however attitudes regarding its value differ by country and decision-making context. Although there are some collaborations in the field of RWE, such as the GetReal Initiative and European Health Data and Evidence Network, regulators have multi-faceted concerns about challenges from the heterogeneity of European healthcare systems and the different populations, resource levels, financing priorities, settings and delivery, and culture. These factors impact both how healthcare data are collected and managed and the content and quality of the data.^28^ Other fields such as infectious diseases which also rely on RWD have created a framework for its use, for example, the ‘fitness-for-purpose’ models by the COVID-19 Multi-Model Comparison Collaboration.^29^ In Asia, Singapore and Indonesia are the few jurisdictions that explicitly mention the use of RWD in their HTA decision-making process.^30^ It will be useful for other jurisdictions to update their HTA process and methods guide to explicitly incorporate recommendations on the use of RWD/RWE in HTA to guide analysts and companies that are preparing evidence submissions about whether they should invest in the collection and analysis of RWD. Building on the existing framework,^31^ we highlight how such a process can be integrated into current HTA decision-making.

**Figure 2:**
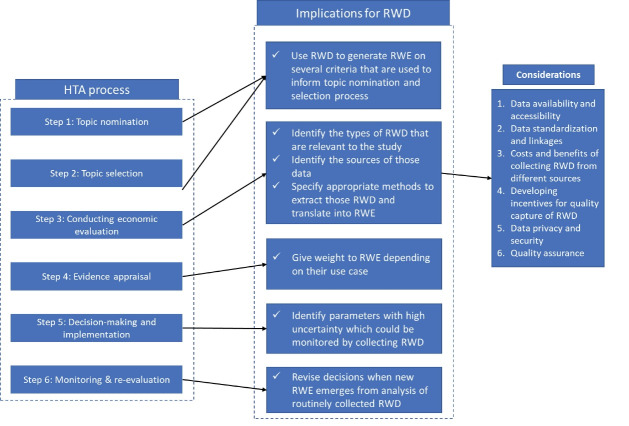
Health Technology Assessment Process and Implications for Real-World Data. Abbreviations: RWD, real-world data; HTA, health technology assessment; RWE, real-world evidence.

 The process, methods, and principles of making evidence-informed decisions using HTA in healthcare have been well-established.^24^ Generally, this process includes 6 steps: (*i*) topic nomination, (*ii*) topic prioritisation and selection, (*iii*) conducting economic evaluation, (*iv*) evidence appraisal, (*v*) decision-making and implementation, and (*vi*) monitoring and evaluating the outcomes of decisions. Different stakeholders participate at different stages of this process including decision-makers, HTA agencies, researchers, clinical experts, patients, and the private sector (Figure 2).

 The discussion of RWD can begin at steps 1 and 2 (topic nomination, and topic selection), where stakeholders nominate topics for evaluation and HTA agencies prioritise and select topics before conducting an HTA. Topics are usually prioritised and selected based on several criteria including burden and severity of disease, safety and effectiveness of health intervention, variation in practice, economic implications on household expenditure and payer’s budget, and equity, ethical and social considerations, feasibility, etc.^32^ Research on these criteria relies on the use of RWD (as RCTs may not be appropriate) and therefore, RWE can inform topic selection decisions.

 In step 3 (conducting economic evaluation), researchers and HTA agencies can identify: (*i*) preferences or the need to include RWD, availability, and acceptance of RWD that are relevant to the study. Information related to the disease context, patient population, intervention and comparator, long-term safety, patient reported outcomes, and costs are usually where RWD may be used. While RCTs remain the gold standard, RWD can supplement or be used when RCTs are not available (or RCTs do not adequately represent target population) for key parameters such as long-term safety and effectiveness (real-world effectiveness). The ‘value-of-information analysis’ framework offers one approach to decision-making, for when and what types of data to collect^33^; (*ii*) upon establishing the types of RWD to collect, researchers can engage with HTA agencies, clinical experts, and the private sector to identify different sources of those data. This engagement may allow researchers to access wider sources of RWD. Evidence costs money, therefore, a balance should be struck between the relevance of a new registry (or any other data) in relation to the burden of collection. The expected benefits and improvement from new evidence is weighed against the cost of the information.^34^ Any consideration of patient medical records as a public good, calls into question the safety and security of individual data. Hence, we recommend that jurisdictions comply with their national guidelines but at the same time, promote active discussion of the tensions between access to RWD and adequate data protection, to arrive at a compromise between these two needs; (*iii*) HTA agencies can specify and guide researchers on the methods that are appropriate while extracting and translating RWD into RWE.^14,35^ While many perceive observational approaches as lacking credibility, researchers can work to overcome these concerns and improve the overall rigor of such studies. The use of checklists for good reporting practices^14^ is strongly encouraged, and submission of completed checklists is now required by some journals to validate manuscripts. Another way to increase credibility is by publishing detailed protocols of real-world studies in a public and online repository. Study registration, particularly for RWD studies intended to formally test hypotheses around comparative effectiveness, has been proposed by the Professional Society for Health Economics and Outcomes Research and International Society for Pharmacoepidemiology joint task force to improve transparency and trust in RWE.^1^ This may be communicated through consultation or by adding information on their national HTA methods guidelines. Such purposive data collection processes, where stakeholders enter binding agreements, has been shown to overcome challenges associated with using RWD.^36^

 In step 4 (evidence appraisal), HTA agencies may consider giving weight to RWE before making recommendations to decision-makers. This may be informed by the choices made when designing the study in step 3, ie, why was RWD sought in the first place (to supplement the RCT data, absence of data from RCTs, preference to use local and long-term data), what types of RWD were used, what were their sources (wearables versus EMRs, public versus private, etc), and methods used to translate RWD into RWE. Specific guidelines on asserting value to RWE produced using this approach are available.^31^

 In step 5 (decision-making and implementation), HTA agencies should inform decision-makers about the uncertainty (exact parameters contributing to such uncertainty are usually communicated to HTA agencies by HTA researchers) attached to their recommendation. Together, researchers, HTA committees, and decision-makers, should make a commitment to monitor those parameters by identifying which RWD and data source they correspond to, for example, utilities or PROs which may be collected from EMRs. Decision-making approaches such as managed entry agreements are already practicing this process of monitoring key parameters and the benefits are evident.^37^

 Finally in step 6 (monitoring & evaluating decisions), HTA agencies may be able to track the impact of and amend their initial decision based on new RWE that emerges from analysis of RWD for parameters (with high uncertainty at the time of the initial decision) identified in step 5. This often-neglected step of monitoring and re-evaluation is becoming an increasingly important area of concern due to issues such as overdiagnosis, overtreatment, and use of low-value care.^38^ They result in a range of downstream consequences such as increased cost to patients and health systems, potential toxicities to patients, diagnosis induced anxiety or depression, etc.^38^ This issue was also raised in the public feedback^16^ on the REALISE guidance document. However, this presents an opportunity for health systems to ‘reset’ and further enhance efficiency. The case study of ‘observational RWE updated’ cost-effectiveness assessment of prophylactic treatment for hereditary angioedema,^39,40^ where the initial decision was updated based on the use of RWD and RWE, presents practical lessons on how steps 5 and 6 may be implemented. Furthermore, the case study of surgery for appendicitis in Thailand during COVID-19 (what type of RWD – hospitalisation due to appendicitis surgery; where were they collected from – national hospital database for patients enrolled under the universal coverage scheme, how were they translated into evidence – by conducting time series analysis of hospitalisation data before and during COVID-19) highlights the overuse of this service for several contextual reasons.^41^ Collection and analysis of RWD enables us to identify such inefficiencies resulting from past decisions and offer opportunities to address them. However, implementing step 6 requires consideration around availability of resources and time for collecting RWD and conducting the re-evaluation, and the appropriate timeframe for re-evaluation.^40^ Stakeholder consultation is advised to find consensus on these issues. This process of explicitly bringing RWD and RWE into reimbursement decisions is summarised in Figure 2. This sub-process (of overall HTA process) for using RWD should be governed by the same principles of the overall HTA process which include participation, transparency, reliability (quality), and accountability.^24^ Similar approaches (albeit not explicitly linking with existing HTA processes) with details on considerations for different stakeholders have been recommended in the literature.^31^ We recommend HTA agencies to include the process of using RWD into HTA (depicted in Figure 2) in their next editions of their national HTA process guidelines.

 Outside the realm of reimbursement decisions, broader health systems or service-related research are regular consumers of RWD and generators of RWE. These include pandemics (real-world effectiveness of vaccines,^42^ effectiveness of non-pharmaceutical interventions,^43^ monitoring and projecting resource needs^43^ such as intensive care unit beds, ventilators, etc, and adherence to policies^43^), measuring health equity,^44^ mental health,^45^ access to care,^46^ or even health systems efficiency.^47^ The primary reason for using RWD in these areas is because conducting RCTs is not ethically or methodologically appropriate.^13^ Future studies could explore how RWD are defined in other research contexts (within and outside the health sector), their types, sources, and methods used to translate into RWE. For example, the term “big data” is also RWD but larger in volume and scope, expanding its use to multiple sectors.^48,49^ Comparing the processes and methods used by different fields while using RWD to generate RWE may help draw out lessons. Furthermore, using a common vocabulary (RWD and RWE) in all research fields may help promote an understanding of their role and acceptance in decision-making. Strengthening the system, process, and methods of collecting and using RWD to inform policies presents benefits to wider health research communities.

###  Limitations

 REALISE meetings were convened in 2019, therefore, some of the information we present in our results may be outdated, especially on the types and sources of RWD used by REALISE members. The need to resort to digital solutions such as telemedicine during the COVID-19 pandemic and accelerating momentum in the digitisation of healthcare systems may have increased the use of RWD sources such as EMRs and improved data linkages. While we have tried to include as many jurisdictions as possible when developing the REALISE guidance, given the varying level of maturity of the health systems and HTA processes across the region, our findings may not be completely applicable or generalisable to the whole of Asia. This is heightened by the lack of, and difficulties in establishing universally accepted methodological standards or principles for, the design, conduct, and reporting of RWE.^50^ In addition, our search of literature was pragmatic informed by the knowledge of existing guidance, hence, our search may not be comprehensive. Nevertheless this paper and the REALISE guidance document should serve as helpful resources for HTA systems in Asia that are seeking to incorporate RWD and RWE in their evidence evaluations and healthcare decision-making.

## Conclusion

 RWD continues to play a significant role in informing decision-making for health globally and notably in Asia despite several barriers. The COVID-19 pandemic has accelerated the use of digital technologies and offers a unique opportunity to expand the use of RWD. The REALISE guidance document serves as one of the repositories of knowledge when considering the use of RWE to inform reimbursement decisions in Asia. RWD deserves its recognition in the current HTA decision-making process, and we call on all stakeholders to contribute to realising its potential going forward.

## Acknowledgements

 Juliet Eames, Gunjeet Kaur and Waranya Rattanavipapong from Health Intervention and Technology Assessment Program, Ministry of Health, Thailand who helped with note-taking during the meeting. National Evidence-based healthcare Collaborating Agency, Korea for supporting the logistics of organizing the first REALISE meeting and Secretariats of HTAsiaLink and National Evidence-based healthcare Collaborating Agency for facilitating communications with HTAsiaLink members.

 As well as Yu Ting Chen, Brandon Chua, Hui Lan Chua, Lou Jing, Siobhan Li, and Wang Yi from the Saw Swee Hock School of Public Health, National University of Singapore (NUS), who helped with facilitating, note-taking and timekeeping during the second REALISE meeting.

## Ethical issues

 This research did not require an ethics approval since the study does not involve human research subjects. Ethics approval was not sought as data was collected from members who had consented to be part of the initiative. In addition, the survey responses were anonymised. The questions and discussions were not harmful to those involved.

## Competing interests

 All support for the present manuscript came from an unrestricted grant from The International Decision Support Initiative (iDSI, https://www.idsihealth.org) [grant number: OPP1134345]. In addition, the grant has covered the attendance of meetings and travel by the corresponding author. The corresponding author has disclosed all the information related to the current manuscript in the ICMJE Disclosure Form and has no relationships/activities/interests to declare related to the content of the manuscript.

## Authors’ contributions

 Preparation of first draft, review, and editing of subsequent drafts, approval of final submitted version: SKC and LWL. Conceptualized and designed the study, project management, supervised data collection, and data analysis, critically revised the first draft and approved the final version: WI and HLW. Review and editing of subsequent drafts and Approval of final submitted version: All authors.

## Data availability statement

 The underlying data is part of the article itself. In addition, this data has been published in the REALISE Guidance document, as well as in the *International Journal of Technology Assessment in Health Care* (IJTAHC).

## Funding

 This work was supported by an unrestricted grant from The International Decision Support Initiative (iDSI, https://www.idsihealth.org) [grant number: OPP1134345], a global network of health, policy and economic expertise, working to achieve universal health coverage and the health Sustainable Development Goal (SDG 3), and which supports countries to get the best value for money from health spending. iDSI receives funding support from the Bill & Melinda Gates Foundation, the UK Department for International Development, and the Rockefeller Foundation. Under the grant conditions of the Bill & Melinda Gates Foundation, a Creative Commons Attribution 4.0 Generic License has already been assigned to the Author Accepted Manuscript version that might arise from this submission. Ryota Nakamura was also supported by the Japan Society for the Promotion of Science KAKENHI (grant number: 18H00862). The funders had no role in study design, data collection and analysis, decision to publish, or preparation of the manuscript. The findings, interpretations and conclusions expressed in this article do not necessarily reflect the views of the aforementioned funding agencies. A representative from iDSI participated in the first REASLISE working group meeting as an observer.

## Supplementary files


Supplementary file 1. Real-World Data Sources Available in Asia.
