# Supplementary material for: What, Where, and How to Collect Real-World Data and Generate Real-World Evidence to Support Drug Reimbursement Decision-Making in Asia: A reflection Into the Past and A Way Forward
Source: Int J Health Policy Manag. 2023 Mar 6;12:6858. doi: 10.34172/ijhpm.2023.6858 (PMC10461954; doi:10.34172/ijhpm.2023.6858)
Supplement: Supplementary file 1 — Real-World Data Sources Available in Asia. [file ijhpm-12-6858-s001.pdf]

Article title: What, Where, and How to Collect Real-World Data and Generate Real-World Evidence to Support Drug Reimbursement Decision-Making in Asia: A reflection Into the Past and A Way Forward

Journal name: International Journal of Health Policy and Management (IJHPM)

Authors' information: Sarin Kc[1], Lydia Wenxin Lin[2], Diana Beatriz Samson Bayani[2], Yaroslava Zemlyanska[2], Amanda Adler[3], Jeonghoon Ahn[4], Kelvin Chan[5,6,7], Dechen Choiphel8, Anne Julienne Genuino-Marfori[9], Brendon Kearney[10,11], Yuehua Liu[12], Ryota Nakamura[13], Fiona Pearce[14], Shankar Prinja[15], Raoh-Fang Pwu[16], Arsul Akmal Shafie[17], Binyan Sui[12], Auliya Suwantika[18], Sean Tunis[19], Hui-Min Wu[16], John Zalcberg[20,21], Kun Zhao[12], Wanrudee Isaranuwatchai[1,22,23], Yot Teerawattananon[1,2], Hwee-Lin Wee[2,24]*

[1]Health Intervention and Technology Assessment Program (HITAP), Ministry of Health, Nonthaburi, Thailand.
[2]Saw Swee Hock School of Public Health, National University of Singapore (NUS), Singapore, Singapore.
[3]The Oxford Centre for Diabetes, Endocrinology, and Metabolism, University of Oxford, Oxford, UK.
[4]Ewha Womans University, Seoul, South Korea.
[5]Sunnybrook Odette Cancer Centre, Toronto, ON, Canada.
[6]Sunnybrook Research Institute, Toronto, ON, Canada.
[7]Canadian Centre for Applied Research in Cancer Control, Toronto, ON, Canada.
[8]Essential Medicine and Technology Division, Department of Medical Services, Ministry of Health, Thimphu, Bhutan.
[9]Health Technology Assessment Unit, Department of Health, Quezon City, Philippines.
[10]Faculty of Medicine, University of Adelaide, Adelaide, SA, Australia.
[11]Health Policy Advisory Committee on Technology, Brisbane, QLD, Australia.
[12]China Health Technology Assessment Centre, National Health Development Research Centre, Ministry of Health, Beijing, China.
[13]Hitotsubashi Institute for Advanced Study, Hitotsubashi University, Tokyo, Japan.
[14]Agency for Care Effectiveness, Ministry of Health, Singapore, Singapore.
[15]Department of Community Medicine and School of Public Health, Post Graduate Institute of Medical Education and Research, Chandigarh, India.
[16]Taiwan National Hepatitis C Program Office, Ministry of Health and Welfare, Taipei, Taiwan.
[17]Discipline of Social and Administrative Pharmacy, School of Pharmaceutical Sciences, Universiti Sains Malaysia, Penang, Malaysia.
[18]Department of Pharmacology and Clinical Pharmacy, Faculty of Pharmacy, Universitas Padjadjaran, Sumedang, Indonesia.
[19]Center for Medical Technology Policy (CMTP), Baltimore, MD, USA.
[20]Cancer Research Program, School of Public Health and Preventive Medicine, Monash University, Melbourne, VIC, Australia.
[21]Department of Medical Oncology, Alfred Hospital, Melbourne, VIC, Australia.
[22]Centre for Excellence in Economic Analysis Research, St. Michael's Hospital, Toronto, ON, Canada.
[23]Institute of Health Policy, Management and Evaluation, University of Toronto, Toronto, ON, Canada.
[24]Department of Pharmacy, Faculty of Science, National University of Singapore (NUS), Singapore, Singapore.
(*Corresponding author: weehweelin@nus.edu.sg)

Supplementary file 1. Real-World Data Sources Available in Asia

Table S1: List of real-world data sources available in Asia (non-exhaustive). Adapted from Milea et al. (2015) and supplemented by the REALISE guidance authors.

Country / RWD source	RWD source
**Bhutan**	
• Bhutan cancer Registry<br>• DHIS-2 (District Health Information System)<br>• Mother and Child Health tracking system<br>• Lab Information system<br>• e-BMSIS (Electronic Bhutan Medical Supplies Inventory System)	Registry<br>EMR<br>EMR<br>EMR<br>EMR
**China**	
• Zhongnan Hospital<br>• West China Hospital<br>• Jinhua Municipal Central Hospital database<br>• The 306th Hospital of PLA, Beijing<br>• Chinese People's liberation army general hospital database<br>• Soochow University Affiliates Children's Hospital database<br>• Urban Employee Basic Medical Insurance database (UEBMI) (Tianjin)<br>• UEBMI (Guangzhou)<br>• UEBMI (Hebei)<br>• Urban Resident Basic Medical Insurance database (URBMI) (Guangzhou)<br>• China Health Insurance Research Association (CHIRA) database<br>• Zhongshan Hospital<br>• New Rural Cooperative Medical Scheme (NRCMS)<br>• Electronic Health Records (EHR) system - Minhang, Shanghai<br>• Guangzhao Psychiatric Hospital	EMR<br>EMR<br>EMR<br>EMR<br>EMR<br>EMR<br>Claims database<br>Claims database<br>Claims database<br>Claims database<br>Claims database<br>EMR<br>Claims database<br>EMR<br>EMR
**India**	
• National Health System Cost Database	Claims database
**Japan**	EMR

• Convergence CT Global Research Network (CGRN)<br>• Medical Data Vision (MDV) EBM Provider<br>• Hamamatsu Medical University Database<br>• Osaka University Database Computer Operating System Database Dokkyo Medical University<br>• EMR Retrieval System-Kyoto University Hospital<br>• Nihon University School of Medicine Clinical Data Warehouse<br>• Japan Medical Data Centre (JMDC) Claims database<br>• JammNet<br>• Diagnosis Procedure Code Database<br>• NHI Database<br>• Medi-Trend<br>• Nihon-Chouzai Pharmacy Claim Database<br>• JMIRI Pharmacy Claims Database<br>• Long term care insurance (Kaigo Hoken)	Hospital administration<br>EMR<br>EMR<br>EMR<br>EMR<br>Claims database<br>Claims database<br>EMR<br>Claims database<br>Prescription<br>Prescription<br>Prescription<br>Claims database
**Malaysia**<br>• United Nations University (UNU)-Casemix database<br>• Electronic Health Management Information System<br>• Acute Stroke Registry Malaysia<br>• Malaysian Thalassaemia Registry<br>• National Dermatology Registry<br>• Malaysian Gastrointestinal Registry<br>• Malaysian National Neonatal Registry<br>• National Cardiovascular Disease Database<br>• National Cancer Patient Registry<br>• National Cardiovascular and thoracic surgical database<br>• National Eye Database<br>• National Inflammatory Arthritis Registry<br>• National Neurology Registry	Hospital administration (discharge records)<br>Hospital administration<br>Registry<br>Registry<br>Registry<br>Registry<br>Registry<br>Registry<br>Registry<br>Registry<br>Registry<br>Registry<br>Registry<br>Registry<br>Registry<br>Registry<br>Registry

• National Obstetrics Registry (NOR) Malaysia<br>• National Renal Registry<br>• Malaysian Registry of Renal Biopsy<br>• National Transplant Registry<br>• National Trauma Database<br>• National Orthopaedic Registry Malaysia<br>• National Suicide Registry Malaysia<br>• Malaysian Registry of Intensive Care	Registry<br>Registry<br>Registry<br>Registry
**Philippines**<br>• Philippine Health Insurance Corporation (PhilHealth) Claims Database<br>• Philippine Health Insurance Corporation (PhilHealth) Cost Database for Z-Benefits<br>• Philippine Integrated Disease Surveillance and Response (PIDSR)<br>• Philippine Renal Disease Registry (PRDR)<br>• Field Health Service Information System<br>• National Injury Surveillance System	Claims database<br>Claims database<br>Registry<br>Registry<br>Database<br>Database
**Singapore**<br>• Casemix database<br>• Medisave database<br>• National Electronics Health Records Database<br>• National Immunization Registry<br>• Various condition-specific registries including Singapore Acute Myocardial Infarction Registry, Singapore Cancer Registry, Singapore Diabetes Registry, Singapore Stroke Registry, Singapore Renal Registry, etc.	Hospital<br>Claims database<br>EMR<br>Registry<br>Registry

**South Korea**<br>• National Health Insurance Corporation (NHIC) database<br>• Health Insurance Review and Assessment (HIRA) database<br>• NCI Central Cancer Registry<br>• National Institute of Health Clinical Research Information System (CRIS)<br>• All hospitals have EMR systems compliant to HL7 and connected with PACS and OCS for various research	Claims database<br>Claims database<br>Registry<br>Registry (only publicly funded research registries)<br>EMR (with image data)
**Taiwan**<br>• National Health Insurance (NHI) Database<br>• NHI DAA-treated patients registry<br>• NHI immune oncology drugs treated patient registry<br>• Taiwan Cancer Registry<br>• Cancer screening registries<br>• Adult preventive health information file<br>• Rare disease data<br>• Data of genetic disease<br>• Notifiable disease dataset of confirmed cases<br>• Symptom Surveillance and Reporting System Database<br>• Infectious diseases database (tuberculosis, HIV/AIDS)<br>• Database of National Immunization Information System<br>• Birth certificate application<br>• Survey for three-hypers series<br>• National Health Interview Survey<br>• Chang Gung Research Database (CGRD)<br>• China Medical University Hospital Clinical Research Data Repository (CMUH-CRDR)<br>• National Taiwan University Hospital Integrated Health Care Information System (NTUH-IHIS)	Claims database<br>Registry/Claims database<br>Registry/Claims database<br>Registry<br>Registry<br>Registry<br>Registry<br>Registry<br>Registry<br>Registry<br>Registry<br>Registry<br>Registry<br>Health survey<br>Health survey<br>EMR<br>EMR<br>EMR<br>EMR<br>EMR

• Taipei Medical University Healthcare System Clinical Data<br>• Taipei Veterans General Hospital Big Data Center (Taipei VGH BDC)	
**Thailand**<br>• Universal Coverage Scheme<br>• Civil Servant Medical Benefits Scheme<br>• Social Security Scheme<br>• Ramathibodi Hospital Database<br>• Buddhachinaraj Hospital Database<br>• Sunpasitthiprasong Hospital<br>• Nakhon Thai Crown Prince Hospital	 Claims database<br>Claims database<br>Claims database<br>EMR<br>EMR<br>EMR<br>EMR

Milea D, Azmi S, Reginald P, et al. A review of accessibility of administrative healthcare databases in the Asia-Pacific region. J Mark Access Health Policy 2015;3 doi: 10.3402/jmahp.v3.28076
